# Association of subchondral bone marrow lesion localization with weight-bearing pain in people with knee osteoarthritis: data from the Osteoarthritis Initiative

**DOI:** 10.1186/s13075-021-02422-0

**Published:** 2021-01-19

**Authors:** Koji Aso, Seyed Mohsen Shahtaheri, Daniel F. McWilliams, David A. Walsh

**Affiliations:** 1 Pain Centre Versus Arthritis & NIHR Nottingham Biomedical Research Centre, University of Nottingham, Nottingham, NG5 1PB UK; 2 Department of Orthopedic Surgery, Kochi Medical School, Kochi University, 185-1 Oko-cho Kohasu, Nankoku, 783-8505 Japan

## Abstract

**Background:**

Subchondral bone marrow lesions (BMLs) detected on MRI in knee osteoarthritis (OA) are associated with knee pain. The prevalence and progression of subchondral BMLs are increased by mechanical knee load. However, associations of subchondral BML location with weight-bearing knee pain are currently unknown. In this study, we aim to demonstrate associations of subchondral BML location and size with weight-bearing knee pain in knee OA.

**Methods:**

We analyzed 1412 and 582 varus knees from cross-sectional and longitudinal Osteoarthritis Initiative datasets, respectively. BML scores were semi-quantitatively analyzed with the MRI Osteoarthritis Knee Score for 4 subchondral regions (median and lateral femorotibial, medial and lateral patellofemoral) and subspinous region. Weight-bearing and non-weight-bearing pain scores were derived from WOMAC pain items. Correlation and negative binomial regression models were used for analysis of associations between the BML scores and pain at baseline and changes in the BML scores and changes in pain after 24-month follow-up.

**Results:**

Greater BML scores at medial femorotibial and lateral patellofemoral compartments were associated with greater weight-bearing pain scores, and statistical significance was retained after adjusting for BML scores at the other 4 joint compartments and other OA features, as well as for non-weight-bearing pain, age, sex, and body mass index (BMI) (medial femorotibial; *B* = 0.08, *p* = 0.02. patellofemoral; *B* = 0.13, *p* = 0.01). Subanalysis revealed that greater medial femorotibial BML scores were associated with greater pain on walking and standing (*B* = 0.11, *p* = 0.01, and *B* = 0.10, *p* = 0.04, respectively). Lateral patellofemoral BML scores were associated with pain on climbing, respectively (*B* = 0.14, *p* = 0.02). Increases or decreases over 24 months in BML score in the medial femorotibial compartment were significantly associated with increases or decreases in weight-bearing pain severity after adjusting for non-weight-bearing pain, age, sex, baseline weight-bearing pain, BMI, and BML at the other 4 joint compartments (*B* = 0.10, *p* = 0.01).

**Conclusions:**

Subchondral BML size at the medial femorotibial joint compartment was specifically associated with the severity and the change in weight-bearing pain, independent of non-weight-bearing pain, in knee OA. Specific associations of weight-bearing pain with subchondral BMLs in weight-bearing compartments of the knee indicate that BMLs in subchondral bone contribute to biomechanically induced OA pain.

**Supplementary Information:**

The online version contains supplementary material available at 10.1186/s13075-021-02422-0.

## Background

Knee pain is the major source of disability and reason for hospital visits in patients with knee osteoarthritis (OA). OA knee pain is characteristically experienced during weight-bearing activity, but might also occur at rest on an unloaded joint. Weight-bearing pain indicates biomechanical mechanisms, whereas chemical or neuroplastic mechanisms might dominate in non-weight-bearing pain. Recent accumulating clinical evidence [1–3] indicates that subchondral bone plays a role in generating joint pain in OA. Subchondral bone marrow lesions (BMLs) detected on MRI are increased in people with knee OA compared to non-arthritic controls [1] and are strongly associated with pain. A previous study demonstrated that larger baseline subchondral BMLs were associated with greater baseline knee pain, and increases in total subchondral BML volume were associated with increased knee pain severity [3]. Greater BML scores were associated with weight-bearing pain and less so with non-weight-bearing pain [2].

The exact pathophysiology of subchondral BMLs is still under debate. Histologically, subchondral BMLs are characterized by bone marrow necrosis, trabecular abnormalities, bone marrow fibroses, edema, cellular infiltration, and vascular proliferation [4, 5]. Microarray analysis of subchondral BMLs in OA demonstrated upregulation of genes implicated in neurogenesis, osteochondral turnover, and inflammation [5]. Subchondral BMLs might contribute to OA pain by this generation of chemical factors, sensitizing nerves within the subchondral bone. Sensitized subchondral nerves might be activated by biomechanical forces, for example while standing, walking, or climbing stairs. If so, then BMLs in weight-bearing rather than non-weight-bearing regions would be expected to specifically associate with weight-bearing pain. However, associations of subchondral BML location with weight-bearing knee pain are currently unknown.

OA structural change particularly affects weight-bearing components of the joint and mechanical knee load increases the prevalence and progression of subchondral BMLs [6]. Knee OA is most commonly associated with varus angulation, with increased force and OA structural change affecting the medial femorotibial compartment. Subchondral BMLs present predominantly medially in varus and laterally in valgus lower limbs [7]. Subchondral BMLs typically occur in regions of the joint with the most severe structural change, for example, full-thickness cartilage lesions, and associations between BML scores and cartilage volume were reported [8]. A previous systematic review [6] suggested that a greater medial or lateral load was related to compartment-specific BMLs. Associations between subchondral BMLs, cartilage damage and varus angulation, and between weight-bearing and non-weight-bearing pain, necessitate careful phenotyping of large numbers of individuals in order to explore specific associations between subchondral BMLs in weight-bearing components of the knee and weight-bearing pain. Longitudinal cohorts can help indicate whether specifically localized subchondral BMLs might mediate weight-bearing pain and support the development of BML scores for the stratification of treatments aiming to improve weight-bearing pain.

The purpose of this study was to assess the association of subchondral BML location and size with weight-bearing knee pain using the Osteoarthritis Initiative (OAI) dataset. We hypothesized that subchondral BML size at the medial femorotibial joint compartment in people with varus angulation is associated with severity of weight-bearing pain, independent of non-weight-bearing pain and the other OA-related MRI features, and that increasing subchondral BML size over time is associated with increased weight-bearing pain severity.

## Patients and methods

### Study design and subjects

**Fig. 1:**
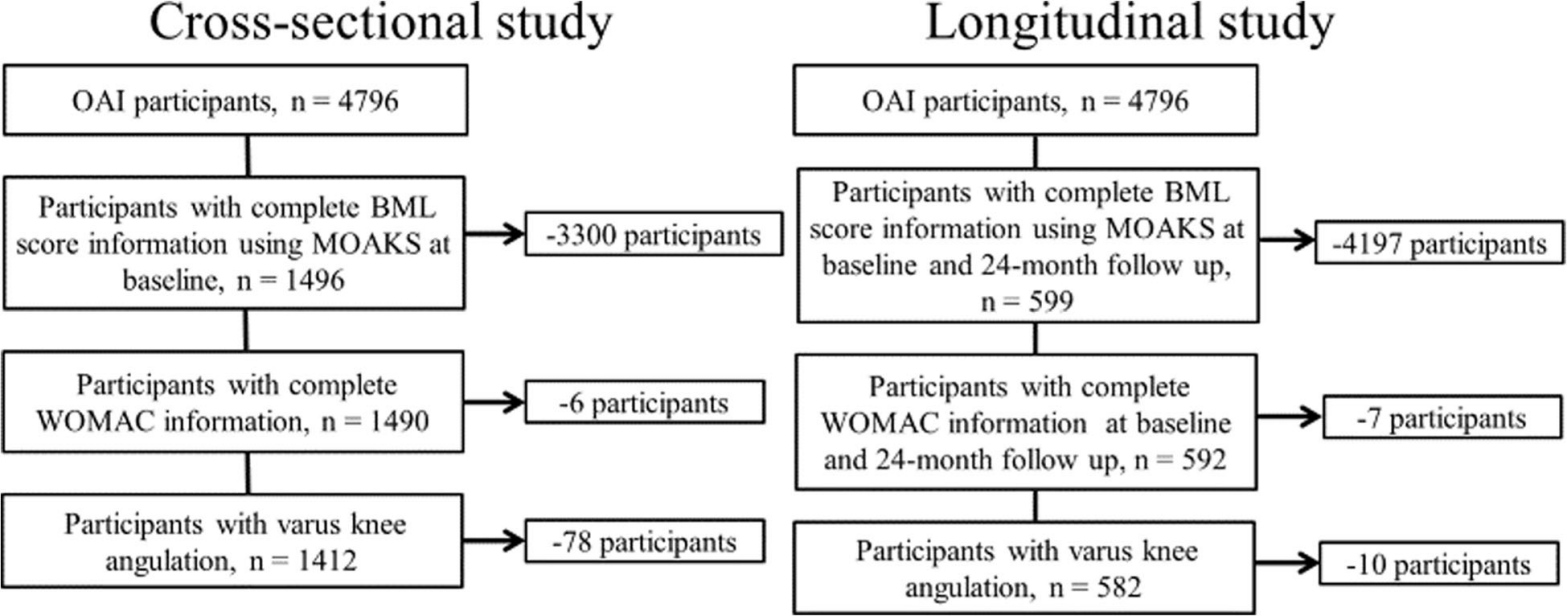
Subject selection from OAI database. *n*, number of participants included in the analysis

Subjects were selected from the OAI, a publicly available multi-center, longitudinal, prospective observational study of knee OA (https://nda.nih.gov/oai). The OAI provides access to the data and images from the OAI longitudinal cohort study and includes data of MRI and radiographic images and Western Ontario and McMaster Universities Osteoarthritis Index (WOMAC) questionnaires [9]. Figure 1 shows a flowchart of participant selection. Of the 4796 participants enrolled in the OAI study, varus knee OA with BML score information of the MRI Osteoarthritis Knee Score (MOAKS) [10], WOMAC questionnaires, femorotibial angle (FTA), and body mass index (BMI) were selected. For subjects with bilateral symptomatic knee OA, one knee was randomly selected using random number generator. We analyzed data from 1412 and 582 knees in the cross-sectional and longitudinal OAI datasets, respectively.

### Knee MRI grading

**Fig. 2:**
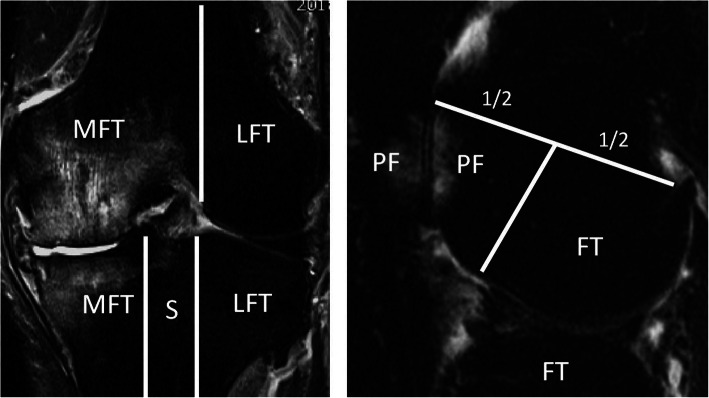
Delineation of subregional divisions for BML scoring. For BML scoring, the knee was divided into 4 articular sub-regions (medial and lateral femorotibial, and medial and lateral patellofemoral compartment) and subspinous region based on MOAKS; MFT, median femorotibial compartment; LFT, lateral femorotibial compartment; PF, patellofemoral compartment; S, subspinous region

We used score data from MRI Osteoarthritis Knee Score (MOAKS) at baseline and 24-month follow-up as knee MRI grading. Intra- or inter-rater reliabilities for subchondral BML score data used in this study were very good to excellent (weighted kappa = 0.76–1.00, 0.76–0.96, respectively) [10]. For subjects who had multiple score data on the same MRI imaging, the median value was used for this study. In MOAKS, subchondral BMLs were scored in each of the 15 anatomical locations according to percentage of the volume of each BML including volume of any associated cysts as 0 = none, 1 < 33%, 2 = 33–66%, and 3 > 66% using the sagittal 3D double-echo steady-state image [10]. In BML scoring for this study, the 15 anatomical locations were integrated into 4 subchondral regions (medial and lateral femorotibial, medial and lateral patellofemoral) and subspinous region (Fig. 2). Articular cartilage, osteophytes, Hoffa’s synovitis, effusion, medial meniscus extrusion, and anterior cruciate ligament tears were scored based on MOAKS (supplemental text) [10]. We used the BML scores in the 5 anatomical regions, total cartilage score (0–42), total osteophyte score (0–32), Hoffa’s synovitis score (0–3), effusion-synovitis score (0–3), medial meniscus extrusion (0–3), and anterior cruciate ligament tears (absent or present).

### Knee pain assessment

Knee pain was assessed at baseline and 24-month follow-up using the WOMAC questionnaires. A recent factor analysis of the five WOMAC pain questions suggested that there are two constructs within the five questions [11]. Based on the result, we defined weight-bearing pain as the sum of three WOMAC questions (pain on climbing stairs, on walking, and on standing) and non-weight-bearing pain as the sum of two WOMAC questions (pain in bed and when sitting or lying). The time points of interest for cross-sectional and longitudinal study were the baseline visit and baseline and 24-month follow-up visits.

### Statistical analysis

Statistical analyses were performed with SPSS version 26 software. Spearman’s correlation coefficients were used for associations between BML score and weight-bearing pain. Pearson’s correlation coefficients were used for associations between changes in BML scores and changes in weight-bearing pain. Spearman’s and Pearson’s correlation coefficients and the *p* values were used for exploratory analysis; therefore, Bonferroni corrections are not used for the correlations. Negative binomial regression was performed for analysis of associations between weight-bearing or non-weight-bearing pain or total WOMAC score and scores of BML, articular cartilage, osteophytes, Hoffa’s synovitis, effusion, and medial meniscus extrusion. *p* < 0.05 indicated statistical significance.

## Results

The participant characteristics in this cross-sectional and longitudinal study are shown in Table 1.

**Table 1:** Participant characteristics at baseline in cross-sectional and longitudinal study

	Cross-sectional study	Longitudinal study
Included participants	1412	582
Male (%)	38.9	42.8
Age (years)	61.7 (8.8)	61.4 (8.8)
BMI (kg/m^2^)	29.6 (4.9)	30.7 (4.8)
Total BML score, 0–45	4.2 (3.7)	3.7 (2.8)
BML score at medial FT joint, 0–15	1.2 (2.0)	1.2 (1.7)
BML score at lateral FT joint, 0–15	0.6 (1.4)	0.2 (0.6)
BML score at medial PF joint, 0–6	0.8 (1.0)	0.8 (1.0)
BML score at lateral PF joint, 0–6	0.9 (1.2)	1.1 (1.5)
BML score at subspinous region, 0–3	0.5 (0.8)	0.4 (0.7)
Articular cartilage score, 0–42	9.0 (5.6)	9.0 (4.7)
Osteophyte score, 0–32	8.8 (6.5)	8.7 (6.4)
Hoffa’s synovitis score, 0–3	0.7 (0.7)	0.7 (0.7)
Effusion-synovitis score, 0–3	0.8 (0.9)	0.8 (0.8)
Medial meniscus extrusion score, 0–3	0.9 (1.0)	1.1 (1.0)
Anterior cruciate ligament tears (%)	9.4	6.2
FTA (degree)	185.9 (2.6)	186.1 (2.0)
KL grade (0/1/2/3/4) (%)	14.0/20.0/32.3/25.3/8.5	0/12.7/51.0/36.3/0
Weight-bearing pain, 0–15	2.3 (2.4)	1.7 (2.1)
Non-weight-bearing pain, 0–10	0.9 (1.5)	0.7 (1.3)

Data of at baseline in cross-sectional and longitudinal study are displayed as mean (SD)

*BMI* body mass index, *BML* bone marrow lesion, *FTA* femorotibial angle, *FT* femorotibial, *PF* patellofemoral, *KL* Kellgren-Lawrence grade

### Cross-sectional analyses

Greater BML, cartilage, osteophyte, Hoffa’s synovitis, or effusion-synovitis scores were each associated with greater weight-bearing pain adjusted for non-weight-bearing pain, age, sex, and BMI (supplemental table). Only association between BML scores and weight-bearing pain remained significant after adjusting for the other OA-related MRI features and anterior cruciate ligament tears (supplemental table). Separate models explored association of BML localization with weight-bearing pain. Greater BML scores at medial femorotibial and lateral patellofemoral compartments were significantly associated with greater weight-bearing pain, and these associations retained significance after adjustment for BML scores at the other 4 joint compartments (Table 2). Subanalysis revealed that greater BML scores at lateral patellofemoral compartments were significantly associated only with greater pain on climbing, whereas the greater scores at medial femorotibial compartments were significantly associated with pain on walking and standing (Table 3).

**Table 2:** Cross-sectional associations between OA-related MRI features and weight-bearing pain

	Spearman’s r	*p*	Β_1_ (95% CI)	*p*	Β_2_ (95% CI)	*p*
Total BML score	0.34	< 0.001**	0.08 (0.04–0.11)	< 0.001**	0.07 (0.02–0.11)	0.005**
Medial FT joint	0.29	< 0.001**	0.10 (0.04–0.16)	0.001**	0.08 (0.02–0.16)	0.02*
Lateral FT joint	0.09	0.003**	−0.01 (− 0.18–0.15)	0.87	− 0.03 (− 0.19–0.14)	0.74
Medial PF joint	0.03	0.35	0.06 (− 0.06–0.17)	0.32	0.06 (− 0.06–0.18)	0.31
Lateral PF joint	0.13	< 0.001**	0.14 (0.05–0.23)	0.003**	0.13 (0.03–0.23)	0.01*
Subspinous region	0.18	< 0.001**	−0.04 (− 0.21–0.14)	0.69	− 0.06 (− 0.24–0.12)	0.53
Articular cartilage score	0.32	< 0.001**	0.05 (0.01–0.1)	0.03*	0.02 (− 0.01–0.05)	0.25
Osteophytes score	0.26	< 0.001**	0.04 (0.01–0.07)	0.01*	0.01 (− 0.01–0.03)	0.46
Hoffa’s synovitis score	0.18	< 0.001**	0.3 (0.03–0.60)	0.03*	−0.03 (− 0.21–0.15)	0.72
Effusion score	0.24	< 0.001**	0.4 (0.13–0.59)	0.002**	0.08 (−0.06–0.22)	0.26
Medial meniscus extrusion score	0.22	< 0.001**	0.15 (−0.05–0.36)	0.14	0.03 (−0.12–0.13)	0.96

B_1_: OA-related MRI features adjusted for non-weight-bearing pain, age, sex, and BMI. BML subscores were in addition adjusted for BML at the other four joint compartments. B_2_: OA-related MRI features adjusted for non-weight-bearing pain, age, sex, BMI, the other OA-related MRI features, and anterior cruciate ligament tears. BML subscores were in addition adjusted for BML at the other four joint compartments.*p < 0.05, ** *p* < 0.01

*BML* bone marrow lesion, *FT* femorotibial, *PF* patellofemoral

**Table 3:** Cross-sectional associations between site-specific BML score and pain on climbing stairs, on walking, and on standing

Site-specific BML scores	Pain on climbing		Pain on walking		Pain on standing	
	Β (95% CI)	***p***	Β (95% CI)	***p***	Β (95% CI)	***p***
Medial FT joint	0.06(−0.02–0.14)	0.13	0.11(0.02–0.20)	0.01*	0.10 (0.04–0.20)	0.04*
Lateral FT joint	0.01(−0.18–0.20)	0.91	− 0.15 (− 0.40–0.11)	0.26	− 0.01 (− 0.26–0.23)	0.93
Medial PF joint	0.13(− 0.004–0.26)	0.06	− 0.07 (− 0.24–0.11)	0.45	− 0.05 (− 0.22–0.13)	0.62
Lateral PF joint	0.14 (0.02–0.25)	0.02*	0.08 (− 0.06–0.22)	0.28	0.12 (− 0.03–0.26)	0.10
Subspinous region	0.03(−0.18–0.23)	0.80	− 0.10 (− 0.35–0.16)	0.46	− 0.20 (− 0.48–0.07)	0.13

B: BML subscores adjusted for BML at the other four joint compartments, non-weight-bearing pain, age, sex, BMI, the other OA-related MRI features and anterior cruciate ligament tears.*p < 0.05

*BML* bone marrow lesion, *FT* femorotibial, *PF* patellofemoral

Greater BML, cartilage, osteophyte, Hoffa’s synovitis, or effusion-synovitis scores were each also associated with greater non-weight-bearing pain adjusted for weight-bearing pain, age, sex, and BMI (Table 4). However, only medial meniscus extrusion score remained significantly associated with non-weight-bearing pain after adjusting for the other OA-related MRI features and anterior cruciate ligament tears (Table 4).

**Table 4:** Cross-sectional associations between OA-related MRI features and non-weight-bearing pain

	Spearmans’ *r*	***p***	Β_1_ (95% CI)	***p***	Β_2_ (95% CI)	***p***
Total BML score	0.19	< 0.001**	− 0.01 (− 0.06–0.04)	0.63	− 0.04 (− 0.10–0.02)	0.21
Articular cartilage score	0.22	< 0.001**	0.01 (− 0.02–0.04)	0.45	− 0.01 (− 0.06–0.04)	0.65
Osteophytes score	0.20	< 0.001**	0.02 (− 0.01–0.05)	0.15	0.02 (− 0.01–0.05)	0.15
Hoffa’s synovitis score	0.11	< 0.001**	0.08 (− 0.16–0.32)	0.52	0.06 (− 0.20–0.31)	0.68
Effusion score	0.18	< 0.001**	− 0.02 (− 0.18–0.18)	0.99	− 0.01 (− 0.004–0.36)	0.90
Medial meniscus extrusion score	0.20	< 0.001*	0.16 (0.01–0.32)	0.04*	0.18 (− 0.55–0.68)	0.045*

B_1_: OA-related MRI features adjusted for weight-bearing pain, age, sex and BMI. B_2_: OA-related MRI features adjusted for weight-bearing pain, age, sex, BMI, the other OA-related MRI features, and anterior cruciate ligament tears. *p < 0.05, ** *p* < 0.01

*BML* bone marrow lesion, *FT* femorotibial, *PF* patellofemoral

### Longitudinal analyses

Changes from baseline to 24-month follow-up of variables in the longitudinal study are shown in Table 5. The increase or decrease in total and site-specific BML scores were associated with increase or decrease in weight-bearing pain severity, respectively. Specifically, the change in BML score at medial femorotibial compartments was significantly associated with change in weight-bearing pain after adjusting for sex, baseline weight-bearing pain, age, BMI, BML at the other four joint compartments, and change in non-weight-bearing pain (Table 6).

**Table 5:** Changes in MRI scores, femorotibial angle, and pain between baseline and 24-month follow-up (*n* = 582)

Characteristic (units or possible range)	Mean change (range)
BMI (kg/m^2^)	0.02 (−10.5 to 13.1)
Total BML score, 0–45	0.7 (−7 to 12)
BML score at medial FT joint, 0–15	0.5 (− 4 to 10)
BML score at lateral FT joint, 0–15	0.1 (− 2 to 5)
BML score at medial PF joint, 0–6	0.03 (− 5 to 3)
BML score at lateral PF joint, 0–6	0.1 (− 3 to 3)
BML score at subspinous region, 0–3	0.07 (− 3 to 3)
Articular cartilage score, 0–42	0.8 (−10 to 9)
Osteophyte score, 0–32	0.4 (− 12 to 14)
Hoffa’s synovitis score, 0–3	0.08 (− 2 to 2)
Effusion-synovitis score, 0–3	0.1 (− 2 to 2)
Medial meniscus extrusion score, 0–3	0.2 (− 2 to 3)
New anterior cruciate ligament tears (%)	0.7
FTA (degree)	0.4 (− 3.9 to 2.0)
Weight-bearing pain, 0–15	0.5 (−10 to 10)
Non-weight-bearing pain, 0–10	0.02 (− 11 to 13)

Positive values represent increases from baseline to follow- up

*BMI* body mass index, *BML* bone marrow lesion, *FTA* femorotibial angle, *FT* femorotibial, *PF* patellofemoral

**Table 6:** Associations between changes in BML scores and changes in weight-bearing pain

BML score changes	Pearson’s r	***p***	Β (95% CI)	***p***
Total score	0.13	0.002*	0.07 (0.00–0.13)	0.04*
Medial FT joint	0.16	< 0.001**	0.10 (0.02–0.18)	0.01*
Lateral FT joint	− 0.06	0.13	− 0.21 (− 0.53–0.11)	0.20
Medial PF joint	− 0.01	0.73	− 0.10(− 0.31–0.11)	0.34
Lateral PF joint	0.02	0.58	0.10 (− 0.09–0.30)	0.29
Subspinous region	0.10	0.01*	0.21 (− 0.03–0.45)	0.09

B_:_ Model for total BML score adjusted for sex, baseline weight-bearing pain, age, and BMI, total BML score, and change in non-weight-bearing pain. BML subscores in addition adjusted for BML at the other four joint compartments. *p < 0.05, ** *p* < 0.01

*BML* bone marrow lesion, *FT* femorotibial, *PF* patellofemoral

No significant longitudinal association between change in medial meniscus extrusion score and change in non-weight-bearing pain was found after adjusting for sex, baseline non-weight-bearing pain, age, BMI and medial meniscus extrusion score, and change in weight-bearing pain (*B* = 0.15 (95% CI, − 0.15-0.44) *p* = 0.33).

## Discussion

In cross-sectional analyses, we firstly demonstrated that subchondral BML size at the medial femorotibial joint compartment in varus knee OA participants was associated with weight-bearing pain severity and that this association was not explained by non-weight-bearing pain, other OA-related MRI features, age, sex, or BMI. In longitudinal analyses, increasing or decreasing subchondral BML size at the medial femorotibial joint compartment was associated with increased or decreased weight-bearing pain severity. Our findings support the hypothesis that subchondral BMLs increase OA knee pain due to biomechanical factors acting through the affected subchondral bone.

Greater BML scores at medial femorotibial compartments were significantly associated with greater weight-bearing pain, especially pain on walking and standing, even after adjusting for non-weight-bearing pain and the other OA-related MRI features. The increase or decrease in BML score at the medial femorotibial compartment was significantly associated with increased or decreased weight-bearing pain severity. In contrast to these findings, BML scores were not significantly associated with non-weight-bearing pain in our fully adjusted models. Hence, we conclude that subchondral BMLs at weight-bearing components are specifically associated with weight-bearing pain. We previously demonstrated that subchondral bone histopathology characteristic of subchondral BMLs, occurring in middle third of medial tibial plateau (an important weight-bearing area), was associated with knee OA pain, not dependent on chondropathy and synovitis [12].

In this study, subchondral BMLs at the lateral patellofemoral joint compartment were also associated with weight-bearing pain, especially pain on climbing. The associations of patellar BMLs with any knee pain and with pain when going up or down stairs have been reported [13]. The patellofemoral joint is one of the most commonly affected compartments in knee OA, and varus knee deformity has been associated with worsening of patellofemoral OA, especially in the lateral facet [14]. We extend these findings to identify that subchondral BMLs at the lateral and not at medial patellofemoral joint compartments were significantly associated with the severity of weight-bearing pain in people with varus angulation. This again suggests a biomechanical explanation linking subchondral BMLs to OA pain. However, patellofemoral BML score change was not associated with pain change, suggesting that subchondral BML changes at the patellofemoral joint has a smaller impact on changes in weight-bearing pain than does subchondral BML change at the femorotibial joint.

Subchondral BMLs represent regions of subchondral bone characterized as displaying increased bone turnover, and expression of factors, including pro-inflammatory cytokines, that can increase nerve sensitization [5]. We previously showed that nerve growth factor expression within osteochondral channels, and subchondral osteoclast density, each is associated with knee OA pain [12], and calcitonin gene-related peptide immunoreactive sensory nerves within osteochondral channels are associated with pain in human and rat knee OA [15]. Activation of sensory nerves in subchondral bone might contribute to weight-bearing pain in knee OA. Subchondral BMLs might be an imaging biomarker for pathology which sensitizes subchondral nerves and therefore increases weight-bearing pain in knee OA. Further investigation is needed to clarify the cellular and molecular factors which might mediate the observed association between subchondral BMLs and weight-bearing pain.

Our results suggested that subchondral BMLs might mediate mechanically induced pain such as during weight-bearing. Biomechanical factors may reciprocally contribute to the pathogenesis of subchondral BMLs. Previous studies [6] reported that increased mechanical load due to malalignment of the knee joint is a risk factor for incident or enlarging subchondral BMLs in the femorotibial joint. Greater BMI that increases the mechanical load on the knee was also associated with increased BMLs size [16]. History of knee injury (e.g., ACL rupture) has been associated with tibiofemoral BMLs [17]. Biomechanical alterations were persistent even after ACL reconstruction [18], and therefore, the altered biomechanical forces might be involved in incident or enlarging subchondral BMLs. Meniscus and cartilage can be shock absorbers that protect subchondral bone from overloading. Associations between meniscal pathology [19, 20] or cartilage loss [19, 21] and increasing subchondral BML size might be mediated by altered biomechanical forces through the subchondral bone. Some have suggested that strenuous physical activity might increase subchondral BML size by repetitive mechanical load on the knee. Strenuous physical activity was associated with increased subchondral BML size [19], but, on the other hand, non-strenuous activity was negatively associated with subchondral BML size [19, 22]. Different levels of physical activity may have different influences on subchondral bone.

Biomechanical unloading by patellofemoral bracing reduced subchondral BML volume at patellofemoral joint compartments in patients with painful patellofemoral OA [23]. However, lateral wedge insoles, which might reduce the load in the medial femorotibial joint compartment during walking [24], did not significantly changes in subchondral BMLs and pain in medial knee OA [25], and the extent of biomechanical unloading that is required to reverse subchondral BML pathology remains uncertain. Treatments with large effects on mechanical load such as high tibial osteotomy and valgus bracing might be needed to reduce subchondral BMLs at medial femorotibial joint compartments.

Pharmacotherapy also has potential to reduce subchondral BMLs. The bisphosphonate zoledronic acid reduced subchondral BML size and knee pain in people with OA [26]. However, recent meta-analysis [27] and randomized controlled trial [28] did not support analgesic effects of bisphosphonates in knee OA. These studies support that biomechanical unloading is more effective than current pharmacotherapy for subchondral BML associated pain. Knee OA pain has multiple sources, and targeting subchondral BMLs would be expected to have greatest benefit in those cases where subchondral BMLs are the predominant driver of weight-bearing pain.

Subchondral BMLs are also found in healthy pain-free young-middle aged adults, with a prevalence of 13–17% [19, 29, 30], which is less than that in symptomatic knee OA. Previous study using animal OA models reported that the first sign of change in the development of OA was subchondral BML which preceded cartilage degeneration [31]. These findings suggest that subchondral BMLs may be involved in the pathogenesis of knee OA before it becomes clinically apparent.

In this study, medial meniscus extrusion score was significantly associated with non-weight-bearing pain severity after adjusting for weight-bearing pain and the other OA-related MRI changes. A recent study reported that patients with medial submeniscal flap tear complained of pains during sleep, but not during daytime activities [32]. Pain associated with meniscal tears might be caused by increased mechanical load on meniscus not only during knee loading but also during unloaded knee flexion-extension motion. However, change in meniscus extrusion score was not associated with change in non-weight-bearing pain, which suggests that meniscus extrusion may have a relative small impact on non-weight-bearing pain.

Not only subchondral BMLs, but also synovitis [33], effusion-synovitis [34], cartilage defects [35], and osteophytes [36] have previously been associated with knee OA pain. We also demonstrated that subchondral BML, osteophyte, and effusion scores were independently associated with total pain score, even after adjusting for the other OA-related MRI features (supplemental table). Associations of pain with cartilage and Hoffa’s synovitis scores did not retain statistical significance after adjusting for the other OA-related MRI features including BML score, suggesting that some of these observed associations may be explained by other closely associated OA features (supplemental table).

Our study has several potential limitations. We investigated only 2 time points (baseline and 24 months). A previous study using OAI dataset demonstrated that changes in total subchondral BML volume after 24 months were positively associated with changes in knee pain severity [3]. However, multiple assessments with shorter intervals are needed to evaluate how fluctuations of subchondral BML size relate to changes in weight-bearing pain. Subchondral BML size was assessed semi-quantitatively and, although our study had a large sample size, quantitative BML measurements might have provided additional information. Direct measurement of mechanical loading through joint compartments was not possible in the current study, and interventional studies would be required to confirm our conclusions that subchondral BMLs are biomarkers for pathology which causes biomechanically induced nociceptive pain.

## Conclusions

We demonstrated that subchondral BML size at the medial femorotibial joint compartment in varus knee OA was associated with weight-bearing pain and that this association was specific for weight-bearing rather than non-weight-bearing pain and was over and above any effects of other OA-related MRI features, age, sex, and BMI. Our findings suggest that specific association of weight-bearing pain with subchondral BMLs in weight-bearing compartments of the knee indicates that subchondral BMLs aggravate biomechanical factors leading to OA pain.

## Supplementary Information

**Additional file 1.**

**Additional file 2.**

## Data Availability

The datasets analyzed during this study are available from the Osteoarthritis Initiative website (https://nda.nih.gov/oai/).
