# Supplementary material for: Association of subchondral bone marrow lesion localization with weight-bearing pain in people with knee osteoarthritis: data from the Osteoarthritis Initiative
Source: Arthritis Res Ther. 2021 Jan 19;23:35. doi: 10.1186/s13075-021-02422-0 (PMC7816469; doi:10.1186/s13075-021-02422-0)
Supplement: Supplementary file 1 — Additional file 1. [file 13075_2021_2422_MOESM1_ESM.docx]

**Supplemental text**

**Semi-quantitative scoring methods of knee MRI using MOAKS** [10]

For this study, the sagittal and coronal intermediate-weighted turbo spin echo sequences, the sagittal 3D double-echo steady-state (DESS) image, and the axial and coronal multiplanar reformats of the DESS were used.

BMLs was scored as described in main text. Cartilage morphology was scored in each of the14 anatomical locations (except the subspinous region) according to size of any cartilage loss as; 0 = none, 1 < 10% of the surface area of the region, 2 = 10-75% of the surface area of the region, 3 > 75% of the surface area of the region. Osteophytes are scored in each of the 12 location according to size as; 0 = none, 1 = small, 2 = medium, 3 = large. Hoffa’s synovitis was scored according to size as; 0 = normal, 1 = mild, 2 = moderate, 3 = severe. Effusion-synovitis was scored based on size of hyper intensity within the articular cavity representing a composite of effusion and synovial thickening as; 0 = none, 1 = small, 2 = medium, 3 = large. Medial meniscus extrusion was scored as; 0 < 2 mm, 1 = 2-2.9 mm, 2 = 3-4.9 mm, 3 > 5 mm. Anterior cruciate ligament tear was scored as either absent or present.
