# Supplementary material for: Association of subchondral bone marrow lesion localization with weight-bearing pain in people with knee osteoarthritis: data from the Osteoarthritis Initiative
Source: Arthritis Res Ther. 2021 Jan 19;23:35. doi: 10.1186/s13075-021-02422-0 (PMC7816469; doi:10.1186/s13075-021-02422-0)
Supplement: Supplementary file 2 — Additional file 2. [file 13075_2021_2422_MOESM2_ESM.docx]

**Supplemental Table** **Cross-sectional associations between OA related MRI features and total pain score**

|  | Spearman’s r | P | Β_1_ (95% CI) | P | Β_2_ (95% CI) | P |
| --- | --- | --- | --- | --- | --- | --- |
| Total BML score | 0.33 | <0.001** | 0.09 (0.06-0.13) | <0.001** | 0.05 (0.01-0.09) | 0.02* |
| Medial FT joint | 0.29 | <0.001** | 0.05 (0.03-0.08) | 0.001** | 0.08 (0.02-0.16) | 0.02* |
| Lateral FT joint | 0.09 | 0.003** | -0.01 (-0.18-0.15) | 0.87 | -0.03 (-0.19-0.14) | 0.74 |
| Medial PF joint | 0.02 | 0.44 | 0.06 (-0.06-0.17) | 0.32 | 0.06 (-0.06-0.18) | 0.31 |
| Lateral PF joint | 0.12 | <0.001** | 0.14 (0.05-0.23) | 0.003** | 0.13 (0.03-0.23) | 0.01* |
| Subspinous region | 0.18 | <0.001** | -0.04 (-0.21-0.14) | 0.69 | -0.06 (-0.24-0.12) | 0.53 |
| Articular cartilage score | 0.32 | <0.001** | 0.05 (0.03-0.08) | <0.001** | 0.003 (-0.03-0.04) | 0.86 |
| Osteophytes score | 0.26 | <0.001** | 0.04 (0.02-0.06) | <0.001** | 0.02 (0.002-0.04) | 0.04* |
| Hoffa’s synovitis score | 0.17 | <0.001** | 0.29 (0.14-0.45) | <0.001** | 0.08 (-0.09-0.26) | 0.35 |
| Effusion score | 0.24 | <0.001** | 0.28 (0.16-0.40) | <0.001** | 0.15 (0.02-0.29) | 0.03* |
| Medial meniscus extrusion score | 0.20 | <0.001** | 0.19 (0.08-0.29) | 0.001** | 0.06 (-0.06-0.18) | 0.36 |

**B_1_:** OA related MRI features adjusted for age, sex and BMI. BML subscores in addition adjusted for BML at the other four joint compartments. **B_2_**: OA related MRI features adjusted for age, sex, BMI, the other OA related MRI features and anterior cruciate ligament tears. *p < 0.05, ** p < 0.01

BML; bone marrow lesion, FT; femorotibial, PF; patellofemoral.
